# Impact of dead time on quantitative ^177^Lu-SPECT (QSPECT) and kidney dosimetry during PRRT

**DOI:** 10.1186/s40658-020-00303-0

**Published:** 2020-05-15

**Authors:** Alessandro Desy, Guillaume F. Bouvet, Andrea Frezza, Philippe Després, Jean-Mathieu Beauregard

**Affiliations:** 1 Cancer Research Centre and Department of Radiology and Nuclear Medicine, Université Laval, Quebec City, Canada; 2 Department of Medical Imaging and Oncology Division of Research Centre, CHU de Québec–Université Laval, 11 côte du Palais, Quebec City, G1R 2J6 Canada; 3 Cancer Research Centre and Department of Physics, Engineering Physics and Optics, Université Laval, Quebec City, QC Canada; 4 Department of Radiation Oncology and Oncology Division of Research Centre, CHU de Québec–Université Laval, 11 côte du Palais, Quebec City, QC G1R 2J6 Canada

**Keywords:** Dead time, Quantitative SPECT, Dosimetry, Radionuclide therapy, ^177^Lu

## Abstract

**Background:**

Dead time may affect the accuracy of quantitative SPECT (QPSECT), and thus of dosimetry. The aim of this study was to quantify the effect of dead time on ^177^Lu-QSPECT and renal dosimetry following peptide receptor radionuclide therapy (PRRT) of neuroendocrine tumours.

**Methods:**

QSPECT/CT was performed on days 1 and 3 during 564 personalized ^177^Lu-octreotate cycles in 166 patients. The dead-time data for each scanning time point was compiled. The impact of not correcting QSPECT for the dead time was assessed for the kidney dosimetry. This was also estimated for empiric PRRT by simulating in our cohort a regime of 7.4 GBq/cycle.

**Results:**

The probability to observe a larger dead time increased with the injected activity. A dead-time loss greater than 5% affected 14.4% and 5.7% of QSPECT scans performed at days 1 and 3, respectively. This resulted in renal absorbed dose estimates that would have been underestimated by more than 5% in 5.7% of cycles if no dead-time correction was applied, with a maximum underestimation of 22.1%. In the simulated empiric regime, this potential dose underestimation would have been limited to 6.2%.

**Conclusion:**

Dead-time correction improves the accuracy of dosimetry in ^177^Lu radionuclide therapy and is warranted in personalized PRRT.

## Background

Peptide receptor radionuclide therapy (PRRT) is an established palliative treatment for patients suffering from neuroendocrine tumours [1]. The widely adopted regime consists of four cycles of 7.4 GBq of ^177^Lu-octreotate. This empiric regime was designed to limit the cumulative absorbed dose to the kidney and bone marrow to 23 and 2 Gy, respectively, in the patient population [2, 3]. However, due to the very high inter-patient variability in the absorbed dose uptake per injected activity (IA) observed in the critical organs [4, 5], personalizing PRRT may be preferable in order to maximize the tumour absorbed dose while limiting that to critical organs. One way to achieve this is to personalize IA based on dosimetry to deliver a prescribed absorbed dose to a critical organ, such as 23 Gy to the kidneys over four induction cycles, which involves IA well above 7.4 GBq in some patients [5, 6]. The accuracy of dosimetry is dependent on that of quantitative imaging. We previously suggested, and recently updated, a practical method for ^177^Lu quantitative SPECT (QSPECT) with a dead-time correction that we implemented for routine clinical dosimetry [7, 8]. Our primary aim was to quantify, in a large cohort of patients undergoing personalized PRRT, the dead time—and the impact of not correcting for it—on the accuracy of ^177^Lu-QSPECT and dosimetry. Secondarily, we wanted to assess the same in the empiric regime of 7.4 GBq/cycle, by simulating the latter in our cohort.

## Materials and methods

### Patients and cycles

A total of 564 ^177^Lu-octreotate cycles (median, 9.1 GBq; range, 0.7–33.7 GBq; Table 1) in 166 consecutive patients enrolled in our prospective clinical trial of personalized PRRT since April 2016 were analysed (NCT02754297) [6]. This included 32 empiric cycles (median, 7.6 GBq; range, 5.6–8.4 GBq) previously administered to 10 of these patients and 532 personalized cycles (median, 9.3 GBq; range, 0.7–33.7 GBq). The institutional Ethics Committee approved the study, and all patients provided a written consent to participate.

**Table 1:** Injected activity, dead time and dosimetry data for personalized and simulated empiric PRRT cycles (*n* = 564)

	Minimum	1st decile	Median	9th decile	Maximum
Personalized PRRT (actual)					
Injected activity (GBq)	0.67	5.39	9.14	17.35	33.67
Dead time					
Day 1 QSPECT (%)	0.44	1.36	2.39	6.17	23.08
Day 3 QSPECT (%)	0.28	0.73	1.36	3.83	22.14
Renal dosimetry					
Absorbed dose (Gy)	0.93	3.58	6.07	8.42	25.89
Absorbed dose per injected activity (Gy/GBq)	0.14	0.34	0.63	1.11	4.93
Deviation without dead-time correction (%)	2.82	− 0.58	− 1.39	− 3.84	− 22.14
Empiric PRRT (simulated, 7.4 GBq/cycle)					
Dead time					
Day 1 QSPECT (%)	0.55	1.06	1.96	4.78	9.82
Day 3 QSPECT (%)	0.28	0.55	1.15	3.08	7.21
Renal dosimetry					
Absorbed dose (Gy)	1.07	2.53	4.64	8.23	36.46
Deviation without dead-time correction (%)	1.90	− 0.54	− 1.04	− 2.60	− 6.65

### QSPECT and dosimetry

^177^Lu-QSPECT/CT was acquired and reconstructed as previously described using a Symbia T6 system (Siemens Healthineers, Erlangen, Germany), with the only modulated parameter being the time per projection (15 s for acquisitions the same day and the day following the injection, and 20 s for following days) [8]. Since photons of any energy can cause dead time, the dead-time correction factor was deduced from the average acquisition wide-spectrum (18–680 keV) counting rate using a lookup table [7, 8]. The dead time corresponds to one minus the inverse of the dead-time correction factor. We used the dead-time constant and calibration factor that were recently obtained (0.55 μs and 9.4 cps/MBq, respectively [8]), which were smaller than those initially estimated (0.78 μs and 10.8 cps/MBq, respectively [7]). Of note, when multiple bed positions were acquired, we considered the dead time of that encompassing the kidney, which was typically the greatest.

Our initial dosimetry protocol was based on a 3-time point QSPECT, at day 0 (~ 4 h), 1 (~ 24 h) and 3 (~72 h) post-injection. Since the day 0 scan contributes little to the accuracy of renal dosimetry, we stopped performing it [9]. Accordingly, dosimetry was computed using only day 1 and 3 scans in the present analysis. Like others, we sampled the activity concentration in tissues using 2-cm spherical volumes of interest [10]. Renal dosimetry was computed by fitting a monoexponential curve, multiplying the area under the time-activity concentration curve by an activity concentration dose factor of 87 mGy g/MBq/h and averaging the absorbed dose of both kidneys [5, 6, 9]. This factor was determined by multiplying the self-absorbed *S* value for the kidney (0.29 Gy/GBq h; OLINDA, Vanderbilt University, Nashville, TN, USA) with the mean kidney volume (300 ml, assuming 1 g = 1 ml). The dead-time loss observed for day 1 and 3 QSPECT, the absorbed dose to the kidney and its deviation without dead-time correction were plotted against the IA. Histograms of the proportion of scans and renal dose estimates that would have deviated by more than 5% or 10% in the absence of dead-time correction were drawn. Graphs and statistics were generated with R (v.1.2.1335; RStudio, Inc., Boston, MA, USA).

### Simulation of empiric PRRT

To estimate the incidence and the impact of dead time in the context of the widely practised fixed-IA ^177^Lu-octreotate PRRT, we simulated an IA of 7.4 GBq for every cycle. The dead-time-corrected kidney activity concentration per IA was multiplied by 7.4 GBq for each imaging time point. We then multiplied the expected wide-spectrum count rate per IA by 7.4 GBq and retrieved the simulated dead-time correction factor from the lookup table. The same analyses described above were then conducted.

## Results

### Dead time and QSPECT

**Fig. 1:**
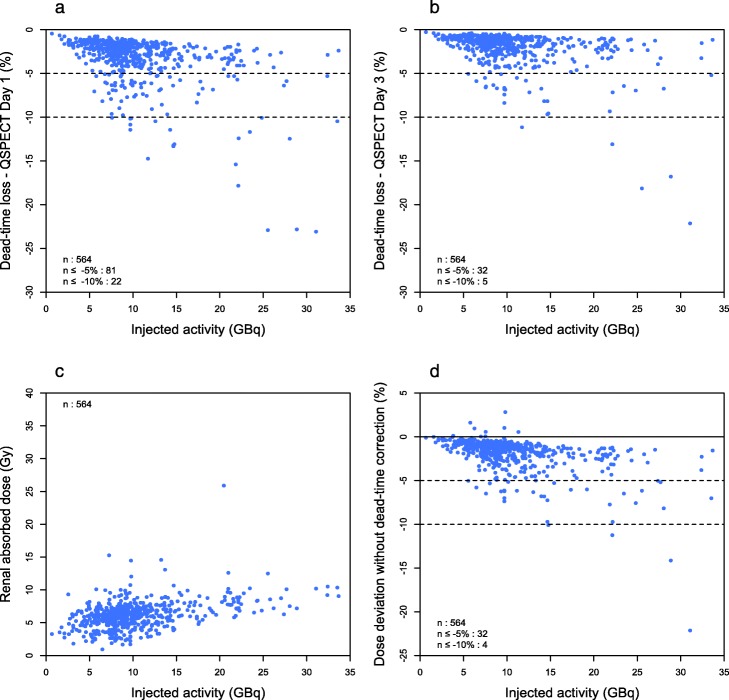
Dead time and renal dosimetry in personalized PRRT. The dead-time count loss affecting day 1 (**a**) and day 3 (**b**) QSPECT scans, the renal absorbed dose (**c**) and its deviation without dead-time correction (**d**) are plotted against the injected activity

**Fig. 2:**
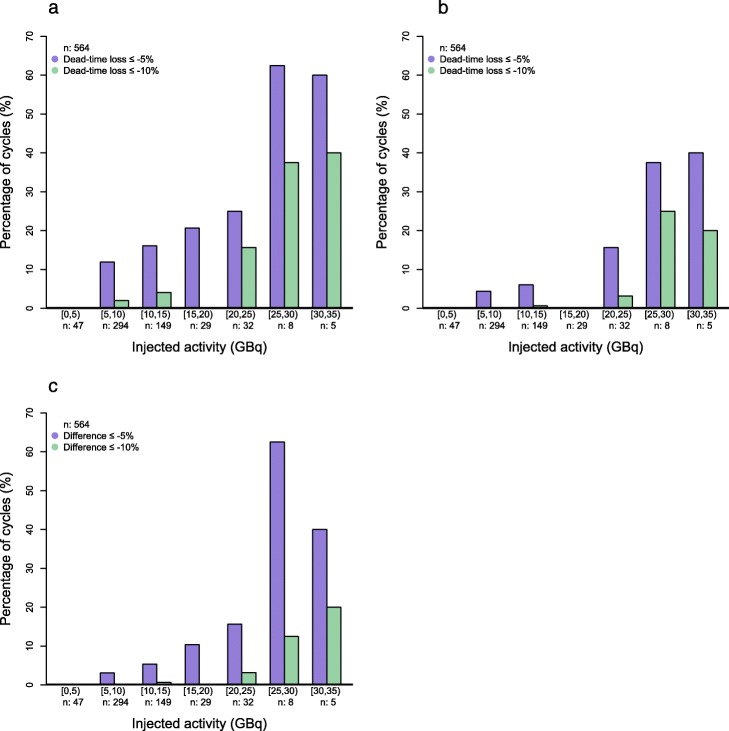
Percentage histograms of cycles per injected activity strata during which the dead time affected day 1 (**a**) or day 3 (**b**) QSPECT, or during which the renal absorbed dose would have been underestimated without dead-time correction (**c**) by at least 5% (purple) or 10% (green)

The median dead time was 2.4% and 1.4% for day 1 and day 3 QSPECT, respectively (Table 1). As expected, the dead time tended to increase with IA and reached up to 23.1% and 22.1%, respectively (Fig. 1a, b). We observed that 14.4% of the day 1 scans (in 23.5% of patients) were affected by a dead-time loss of 5% or more, while at day 3, this figure was 5.7% (in 7.2% of patients). More than 60% of day 1 scans suffered a dead time of at least 5% when the IA was 25 GBq or more (Fig. 2a, b).

### Dead time and dosimetry

**Fig. 3:**
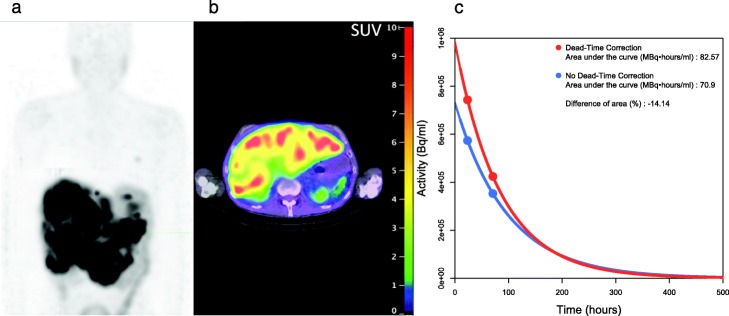
Day3 QSPECT maximum intensity projection (**a**), fused QSPECT/CT slice (**b**) and renal time-activity concentration curve (**c**) of a patient with extensive liver metastases who received 28.9 GBq ^177^Lu-octreotate at his second cycle. The dead time was 22.8% and 16.8% on day 1 and day 3 QSPECT, respectively. The area under the curve–and consequently, the renal absorbed dose – would have been underestimated by 14.1% without dead-time correction

Since our personalized PRRT protocol aims at standardizing the kidney absorbed dose, the latter showed little dependence to IA (Fig. 1c), reflecting the high inter-patient variability in the absorbed dose per IA (Table 1). Not correcting for a dead time would have resulted in a median deviation of the renal absorbed dose by − 1.4%, and the underestimation could get as important as − 22.1% (Table 1). It exceeded − 5% in 5.7% of cycles in 10.2% of patients (Fig. 1d). The probability of a significant impact of dead-time correction on dosimetry increased with IA (Fig. 2c). In 54% of cases involving an IA of 25 GBq or more, dead-time correction avoided an underestimation greater than − 5% to occur. An example of such a case is presented (Fig. 3).

### Simulated empiric PRRT

**Fig. 4:**
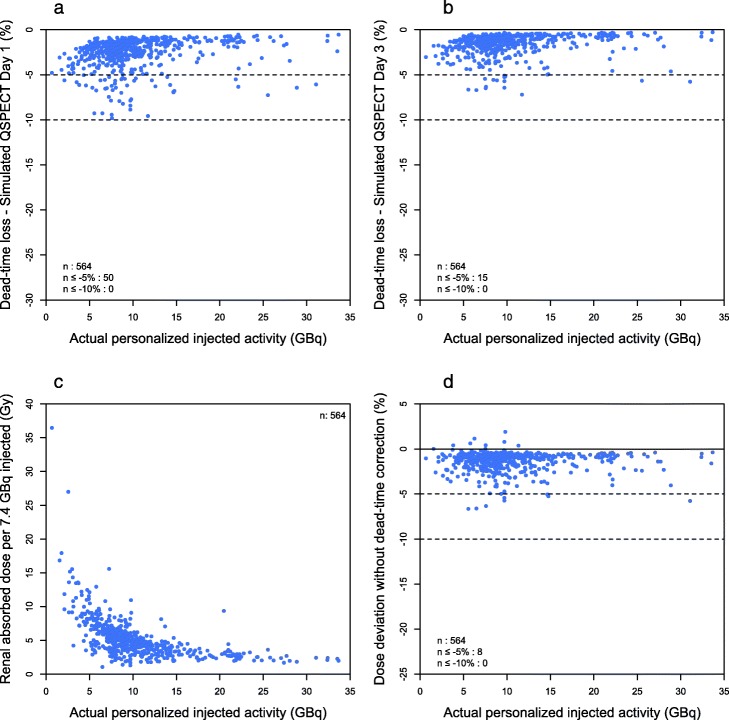
Dead time and dosimetry in simulated empiric PRRT (7.4 GBq/cycle). The dead-time count loss affecting day 1 (**a**) and day 3 (**b**) QSPECT scans, the renal absorbed dose (**c**) and its deviation without dead-time correction (**d**) are plotted against the actual personalized injected activity

If all patients had received 7.4 GBq/cycle, the dead-time loss would not have exceeded 10% at both QSPECT time points (Table 1, Fig. 4a, b). The dead time was at least 5% in 8.9% and 2.7% for day 1 and day 3 scans, respectively (in 13.9% and 4.2% of patients, respectively). Not correcting for dead time would have resulted in an underestimation of kidney absorbed dose of at least − 5% in 1.4% of cycles. Of note, the absorbed dose to the kidney could have gotten as high as 36 Gy/cycle (4.9 Gy/GBq) if PRRT had not been personalized in our cohort (Fig. 4c). The maximum underestimation of renal absorbed dose when not correcting for dead time was − 6.15% (Fig. 4d).

## Discussion

With its low-yield medium-energy gamma emission (208 keV, 11%), ^177^Lu is a therapeutic radionuclide with favourable imaging characteristics. However, our results confirm that dead time, if not accounted for, affects the accuracy of ^177^Lu-QPSECT and consequent dosimetry estimates. The probability of significant dead time increases with the IA, but the level of dead time remains poorly predictable at any IA owing the very high inter-patient variability in ^177^Lu-octreotate retention, which in turn depends on individual factors such as tumour burden and renal function.

As others have found, the impact of dead time is limited when IA does not exceed the widely adopted empiric IA of 7.4 GBq [11, 12]. Nevertheless, simple dead-time correction methods can be easily implemented to maximize the accuracy of QSPECT and remove a layer of uncertainty which would otherwise sum up with many others when performing internal dosimetry [8, 12–14].

The proportion of patients whose dosimetry would be significantly impacted by the lack of dead-time correction is larger in personalized PRRT, which aims to optimize the treatment for each individual. In this regard, a personalized medicine approach is particularly concerned with outlier patients, even if they represent a minority of the population. In PRRT, outliers include patients with a very high tumour burden and consequent retention of ^177^Lu-octreotate combined with a fast activity clearance from the kidneys and other healthy tissues. While these patients are in great need for therapeutic effect, they are undertreated with empiric PRRT. Our personalized PRRT protocol aims to optimize the irradiation of their tumour by personalizing the IA to deliver a standardized renal absorbed dose. The latter would inevitably be exceeded if QSPECT was not dead-time corrected, exposing them to a higher risk of toxicity than intended. Furthermore, at the population level, risk assessment based on non-dead-time-corrected dosimetry data could result in underestimated safety thresholds, or overestimated risk for a given absorbed dose value.

Our results obtained using a SPECT/CT system equipped with NaI crystals are likely valid for other systems having a similar design. However, dead time is expected to be substantially lesser, if not negligible, using a system with pixelized CZT solid-state detectors in the same clinical setting [15].

## Conclusion

For NaI crystal cameras, dead-time correction improves the accuracy of QSPECT and dosimetry in ^177^Lu radionuclide therapy. While dead-time correction is recommended for empiric PRRT, it becomes mandatory in personalized PRRT protocols involving custom IA per cycle. This will likely also apply to other ^177^Lu radionuclide therapies such as the rapidly emerging prostate-specific membrane antigen radioligand therapy.

## Data Availability

Please contact the corresponding author for the data used in this manuscript.

## Abbreviations

CZT: Cadmium zinc telluride
IA: Injected activity
PRRT: Peptide receptor radionuclide therapy
QSPECT: Quantitative single-photon emission computed tomography
